# Gender inequalities in diet quality and their socioeconomic patterning in a nutrition transition context in the Middle East and North Africa: a cross-sectional study in Tunisia

**DOI:** 10.1186/s12937-019-0442-6

**Published:** 2019-03-21

**Authors:** Mohamed Mehdi Abassi, Sonia Sassi, Jalila El Ati, Houda Ben Gharbia, Francis Delpeuch, Pierre Traissac

**Affiliations:** 1 Faculté des Sciences de Tunis, Université Tunis El Manar, 2092 Tunis, Tunisia; 2 INNTA (National Institute of Nutrition and Food Technology), SURVEN (Nutrition Surveillance and Epidemiology in Tunisia) Research Laboratory, 11 rue Jebel Lakhdar, Bab Saadoun, Tunis, Tunisia; 3 IRD (French Research Institute for Sustainable Development), NUTRIPASS Unit, IRD - Université de Montpellier - SupAgro Montpellier, 911 avenue Agropolis, 34394 Montpellier, France

**Keywords:** Gender inequality, Nutrition transition, Diet quality, Diet Quality Index-International (DQI-I), Middle East and North Africa

## Abstract

**Background:**

In a context of nutrition transition and major shifts in lifestyle and diet, the Middle East and North Africa features a marked gender excess adiposity gap detrimental to women. In this setting, where gender issues are especially acute, we investigated gender differences in dietary intake with a focus on diet quality, and how the differences varied with the area of residence and socio-demographic characteristics.

**Methods:**

The study was conducted in 2009–2010 in the Greater Tunis region (Tunisia), as a case study of an advanced nutrition transition context in the region. A cross-sectional survey used a random, stratified, clustered sample of households: 1689 women and 930 men aged 20–49 years were analyzed. Dietary intake was assessed using a 3-day food record. Nutrient content was derived from a specific Tunisian food composition database. We analysed the Diet Quality Index-International (DQI-I) and sub-scores (variety, adequacy, moderation and balance). A score of DQI-I > 60 defined good diet quality. Inequality measures were women vs. men differences in means for interval variables and odds-ratios (OR) for DQI-I > 60. Their variation with socio-demographic characteristics was estimated using models featuring gender x covariate interactions.

**Results:**

Mean energy intake/day was 2300 ± 15 kcal for women vs. 2859 ± 32 kcal for men. By 1000 g/kcal/d women consumed more fruits and sweets but less red meat and soft drinks than men. Women had a higher mean moderation sub-score than men (+ 1.8[1.4, 2.2], *P* < 0.0001) but lower variety (− 2.0[− 2.3, − 1.6], P < 0.0001) and adequacy (− 1.8[− 2.0, − 1.5], P < 0.0001). Thus, the overall mean DQI-I was lower among women than men (58.6 ± 0.3 vs. 60.4 ± 0.3, − 1.8[− 2.6, − 1.0], *P* < 0.0001) as was the proportion of DQI-I > 60 (45.2% vs. 55.7%, OR = 0.7[0.5, 0.8], *P* < 0.0001). Adjusted gender differences in DQI-I decreased with age but were higher in larger households and extreme categories of education (no-schooling and university) vs. the middle categories.

**Conclusion:**

In this nutrition transition context with only average diet quality, it was somewhat lower for women. Socioeconomic patterning of gender contrasts was mild. Beyond, that women had lower adequacy and variety scores but better moderation is a possible pathway for gender specific prevention messages.

**Electronic supplementary material:**

The online version of this article (10.1186/s12937-019-0442-6) contains supplementary material, which is available to authorized users.

## Background

In recent decades, the Middle East and North Africa (MENA) region has experienced a major increase in the prevalence of obesity and nutrition related non-communicable diseases (NCD): the prevalence of obesity and diabetes are now among the highest worldwide [1, 2]. The MENA region is also characterised by major women vs. men contrasts in overweight or obesity as women are about three times more prone to obesity than men [1, 3, 4]. Beyond sex-linked biological differences, a variety of external factors has been put forward to explain this inequality, related to non-egalitarian gender household and social roles in the region [5–8]. In particular, these women vs. men contrasts in obesity have been shown to be much lower in higher socioeconomic strata, likely related with less unequal gender roles [9, 10]. Several hypotheses have been put forward regarding mediating factors at different levels of causation such as slimmer body image models, less sedentary lifestyle or decreased food incentives for women of higher socioeconomic status or who work outside the home. However, detailed evidence for gender differences in lifestyle characteristics is not substantial. Yet, driven by globalisation, socioeconomic changes and urbanisation, major shifts in these lifestyle characteristics are observed in the context of the nutrition transition in middle income countries, and the MENA region is no exception [11, 12]. These especially include dietary shifts away from traditional to more westernised diets with high fat, sugar, and salt contents and an increasing proportion of industrial foods, with contrasted effects on overall diet quality [13].

Tunisia is a country emblematic of the MENA region undergoing the nutrition transition and currently features high prevalences of excess adiposity and nutrition related NCDs, especially in urban areas where obesity concerns a third of the women and about one man out of six [4, 14, 15]. Tunisia has long been one of the most advanced countries of the MENA region regarding gender legislation [16]. However, it still partly shares a core of traditions and social norms with the other countries in the region, somewhat linked to the Muslim culture and which results in unequal gender roles both within the household and in society that are detrimental to women: i.e. non-egalitarian division of household labour, lower expectations regarding education or professional insertion for women, gender constraints on physically active leisure activities or not completely gender neutral legislation. This partly underlies the marked gender inequality in overweight and obesity harmful to women, who, in this context, have been shown to be two to three times more prone to excess adiposity than men [1, 10, 15]. On the other hand, this phenomenon is not exclusive of the persistence of certain types of undernutrition partly linked to micro-nutrient deficiency, e.g. anaemia or iron deficiency, to which women are particularly vulnerable [17].

The objectives of the study were then, firstly to assess gender contrasts in dietary intake among Tunisian adults in a mostly urban nutrition transition context. These contrasts were evaluated from different perspectives including food groups, nutrients and diet quality, using quantitative measures of inequality. Then, by analogy with the inter-sectional approach in the social sciences (which focuses on assessing how gender issues intersects with socio-economic conditions) [5], we assessed variations in gender dietary contrasts according to the demographic and socioeconomic characteristics of the subjects.

## Methods

### Study area

Tunisia is a South-Mediterranean country, with about 11 million inhabitants of which two thirds are urban. According to the Human Development Index, Tunisia ranks 95th out of 175 countries i.e. towards the lower end of the high development group, and ranks 63rd worldwide regarding the Gender Development Index [18]. The study area is the “Greater Tunis” administrative region, which encompasses the four “Governorates” of Ariana, Ben Arous, Manouba and Tunis (the capital city) and includes a quarter of the Tunisian population. Greater Tunis is the most developed region in the country and is mostly urban. It was chosen as a case study of an advanced epidemiological and nutrition transition setting in the MENA region.

### Design and sampling

Data were collected as part of the “Obe-Maghreb” research project, during a cross-sectional survey conducted from March 2009 to January 2010 in the Greater Tunis area and details of the sampling have been previously published [15, 17]. Households were selected using stratified, 2-stage random cluster sampling and all household members aged 6 months to 49 years were included (excluding pregnant women). For this particular study, we analysed the sub-sample of 20–49 year old adults (this age range was originally chosen because the main aim of the “Obe-Maghreb” project was to study the obesity-anemia double burden among women of childbearing age [15, 17]). As reported in a previous publication, the response rate was 89.5% for women and 67.7% for men in this age class. The sample comprised *n* = 1689 women, and *n* = 930 men.

### Measurements and derived variables

#### Gender - sex

The main exposure was the self-reported woman/man variable. It was used to derive women vs. men inequality measures (using man as the reference category) possibly including both sex-linked biological differences and the influence of differential gender roles in the context of the study [6]. In the following, for the sake of readability, we mostly used the word “gender” instead of “women vs. men” (e.g. in “gender differences” or “gender inequalities”), and when appropriate, we discuss whether these contrasts could be partly due to sex related differences (vs. actual gender differences).

#### Socioeconomic and demographic characteristics

Geographic variability was studied as urban vs. rural areas and between the four governorates. Data on age, marital status, as well as details on the level of education and professional occupation of the subject were collected during interviews and recoded for the purpose of analysis (Table 1). Tertiles of an asset-based proxy (computed by multivariate analysis of housing characteristics and ownership of appliances) were used to rank households in increasing welfare categories [15, 19].

**Table 1:** Distribution of sociodemographic factors, by gender, 20–49 y. in Greater Tunis

	Women (*n* = 1651)	Men (*n* = 894)
	%^a^	%^a^
Area	P ^**b**^ = 0.47	
Urban	92.1	92.5
Rural	7.9	7.5
Governorates	P ^**b**^ = 0.84	
Tunis	39.5	39.2
Ariana	23.0	21.4
Ben Arous	22.9	24.1
Manouba	14.6	15.3
Age (years)	P ^**b**^ = 0.54	
20–29	38.6	41.2
30–39	33.0	32.3
40–49	28.4	27.5
Marital status	P ^**b**^ = 0.16	
Married	60.8	57.0
Other	39.2	43.0
Household size	P ^**b**^ = 0.31	
1–3	7.8	9.3
4–5	51.1	51.9
6 or more	41.1	38.8
Education	P ^**b**^ < 0.0001	
No formal schooling	8.8	3.2
Primary school	33.8	33.2
Secondary	35.2	46.4
University	22.2	17.2
Professional activity	P ^**b**^ < 0.0001	
Upper/intermediate	9.9	28.0
Employee/worker	22.5	54.4
Not working/retired	53.3	6.7
Student	14.3	10.9
Household welfare proxy	P ^**b**^ = 0.79	
Lower tertile	33.2	31.9
Intermediate tertile	34.3	35.8
Upper tertile	32.5	32.3

^a^Weighted mean (accounting for unequal probabilities of selection and differential response rates)

^b^*P* value for women vs. men (chi-square test taking into account sampling design)

#### Measurement of dietary intake

Dietary intake was assessed using a 3-day food record (two weekdays and one weekend day) to collect the types and amounts of the meals, foods and beverages consumed [20]. Trained dieticians visited the selected households to give participants detailed instructions on how to record the amount of foods consumed using household tableware. The first step of the 3-day food record was self-administered by the subjects at home. The day of the survey, dieticians reviewed unclear descriptions, errors, omissions, or doubtful entries in the filled pre-printed form and asked the participants to clarify them. For each dish, a list of ingredients, the estimated weight of the raw edible portion and method of preparation were collected from the women in charge of food preparation. The accuracy of portion size of consumed foods was checked using photos of food portions [21] and known weight/specific portions. A list of 203 food items was derived from dietary records (Additional file 1: Table S1).

#### Food groups

**Fig. 1:**
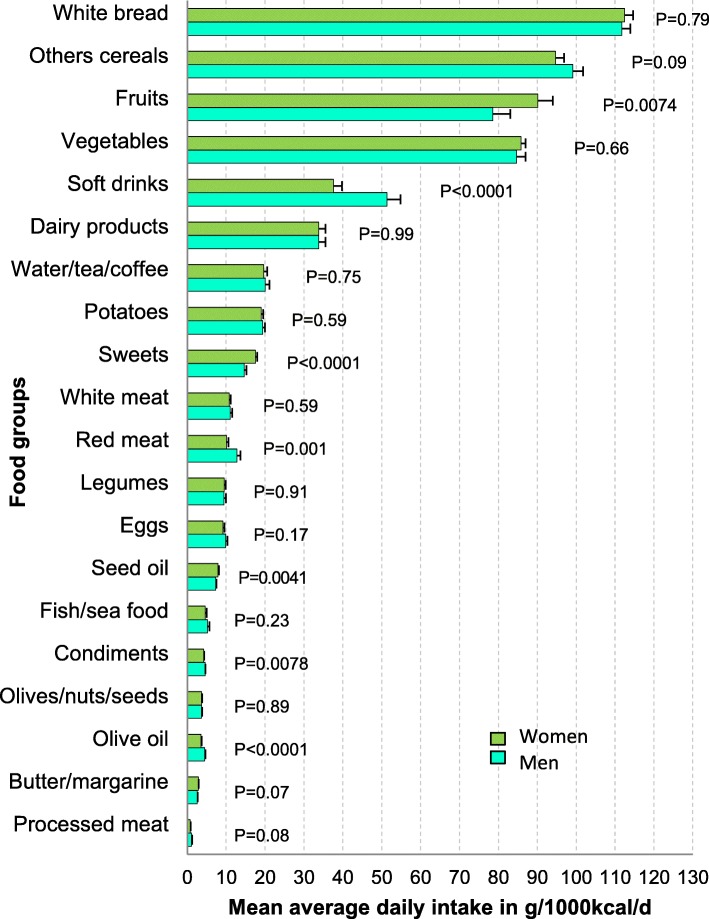
Daily intake of food groups in g/1000 kcal/d, by gender, among 20–49 y., Greater Tunis. Mean average daily intake of food items identified by the 3-day prospective food record (see Additional file 1: Table S1), recoded in 20 food groups (women *n* = 1651, men *n* = 894). Bar is weighted mean value in g/1000 kcal /d for each gender, symbol on bar is standard error of the mean (taking into account complex sampling design). *P*-Value: gender contrast for average daily intake of each food group

Food items were regrouped into 20 food groups (Fig. 1, Additional file 1: Table S1) based on Tunisian food habits and a Mediterranean diet pyramid [22]. Intakes of the different food groups where expressed either in g for absolute values or in g/1000 kcal (i.e. g/4180 kJ) according to the nutrient density model [23].

#### Energy, macro- and micronutrients

The nutritional profile of the recipes was calculated by applying yield factors to the edible parts of raw ingredients to account for the change in weight during cooking, and retention factors to account for changes in their nutritional content [24]. The Tunisian food composition table [25], supplemented by the US Department of Agriculture table [26], additional laboratory analyses and the Food Processor software, version 8.3 [27] were used to estimate the energy and nutritional content (macro- and micronutrients) of identified food items and recipes. Individual energy and nutrient intakes were then derived. Implausible energy intakes were defined as < 500 or > 3500 kcal/d for women and < 1000 or > 4000 kcal/d for men [28]. Energy as a % of requirement was estimated using the FAO (Food and Agriculture Organization of the United Nations) physical activity recommendations for middle income countries [29] and by computing the basal metabolic rate from Henry’s predictive equation [30]. Adequacy of nutrient intakes was assessed with respect to WHO/FAO recommendations [31, 32]. Nutrients were expressed per 1000 kcal [23].

#### Diet quality index

Diet quality was assessed using the Diet Quality Index-International (DQI-I) which is a composite index accounting for the overall quality of the diet by incorporating both nutrient and food group intakes [33]. The overall (0–100) score is the sum of four components variety, adequacy, moderation and balance. Variety (0–20) accounts for the diversity of individual diets at the global level but also the diversity of protein sources. Adequacy (0–40) scores the compliance with given recommendations: we used the Kim & al. [33] threshold values except for vitamin C, iron and calcium, for which we used the WHO/FAO recommendations [32]. The recommended intakes of grains, fibre, fruits and vegetables was dependant on energy intake. Moderation (0–30) focuses on nutrients, expressed as a percentage of energy intake, whose excess intakes are assumed to increase the risk of NCDs. Balance (0–10) assesses whether the percentage of energy provided by carbohydrates, proteins and lipids is suitable and that the ratios of monounsaturated and polyunsaturated fats to saturated fats are balanced. For some of the items in the variety and adequacy components, food group intake was converted into number of servings: data from the Mediterranean diet pyramid [22] and the French National Nutrition and Health Program (PNNS) [34] were used to estimate serving sizes. The global DQI-I score for each subject is the sum of the values of the four components resulting in a score on a 0–100 scale (0 being the lowest and 100 the highest diet quality). The five resulting interval variables (DQI-I and its four sub-components) were used as the main outcomes in this study. However, in specific analyses we also used interval variables DQI-I and sub-components rescaled to % of maximum achievable score (for each subject the value of each sub-score was divided by 20, 40, 30 and 10 for variety, adequacy, moderation and balance respectively). A good quality diet was defined by a DQI-I > 60 [33] and studied as a binary outcome. We then defined binary variables coding for each of the four sub-components > 60% of maximum achievable score by analogy with the cut-off for the overall score.

One of the motivations behind our choice of the DQI-I was that beyond the uni-dimensional assessment of overall diet quality by the total score, its sub-components enabled us to focus on four different dimensions of diet, which are not always correlated, especially in the context of the nutrition transition. Indeed, worldwide, there seem to be trends towards increases in the consumption of both “healthy” and “unhealthy” food items in middle income countries [13]. Concerning the specific case of the DQI-I in our context, a study of Tunisian adolescents also showed contrasted aspects of their diet, depending on which component of the DQI-I was taken into account [35].

### Data management and statistical analysis

Data entry, including quality checks and validation by double entry was performed using EpiData version 3.1 [36] and Stata [37] was used for data management and analysis. All analyses were performed on the complete case sub-sample of women and men after exclusion of subjects with missing values for socioeconomic data, dietary intake and of those with implausible energy intake values (defined above). Sample stratification, clustering and weights (including sampling weights and post-stratification on sex, age and place of residence) were taken into account for all analyses using *svy* Stata commands dedicated to the analysis of data from complex samples [38]. All results are presented as estimates ± design based standard error and/or 95% confidence interval [in brackets]. The type I error risk was set at 0.05 and 0.20 for interactions [39].

As gender was the main exposure studied, gender contrasts were assessed as women minus men difference of means for interval variables (e.g. food groups in g/1000 kcal, nutrients, DQI-I and its four components) or women vs. men OR (Odds-Ratio) for binary variables (e.g. good quality diet defined as DQI-I > 60).

Overall gender contrasts were assessed in models including only the variable coding for gender (with men as the reference category) as independent variable: general linear models for interval response variables (in this case, the regression coefficient is the women vs. men difference of means) and logistic regression for binary variables (the exponentiated regression coefficient is the women vs. men Odds-Ratio).

Inter-sectional gender analysis in the social sciences [5], aims at understanding how gender intersects with socio-economic conditions. By analogy, to study the possible differential association of gender with diet in different socioeconomic conditions, we studied variations in gender contrasts with socio-demographic characteristics of the subject and household. This was achieved by including the gender binary variable x covariate interaction terms in the models [10, 15]. In the first step, separately for each socio-demographic covariate, we fitted a model including the gender binary variable, the covariate and gender binary variable x the covariate interaction as regressors (crude difference or crude odds ratio in Table 4). Next, we fitted one complete adjusted multivariate model including the gender binary variable, the main effects of all covariates and all the interactions gender x covariates (adjusted difference of adjusted odds ratio in Table 4). Adjusted gender contrasts by category of the covariates were estimated based on marginal estimates of the response variable computed at the mean value of all other covariates using the *margins* command in Stata [40].

Gender diet quality contrasts were adjusted for energy intake (kcal/d) in all analyses**.**

## Results

### General characteristics of the sample

Of the 2619 subjects surveyed, [15], 27 were excluded for lack of dietary intake and 47 for implausible energy intake values. Finally 2545 subjects (women *n* = 1651, men *n* = 894) were included in complete case analyses. The majority of subjects lived in urban areas. Mean age was 33.9 ± 0.3 years and two thirds of the subjects were married. There were no gender differences for area, governorate of residence, age, marital status or household wealth score. Gender contrasts regarding education were mild. There was a marked gender contrast for professional occupation: e.g. half the women vs. less than a tenth of the men were not professionally occupied, and one woman out of ten vs. about a third of the men were in the upper/intermediate category (Table 1).

### Dietary intake and diet quality of 20–49 year old adults

Overall, the food group most frequently consumed was cereals (540.3 ± 6.2 g) of which white bread accounted for more than half (287.1 ± 4.7 g), and pasta (94.5 ± 2.5 g). Fruits and vegetables (431.1 ± 9.5 g) and to a lesser extent, potatoes (49.0 ± 1.1 g) were also staples. Total white and red meat mean consumption was 58.1 ± 1.6 g, i.e. less than dairy products (86.8 ± 3.4 g). Consumption of food groups with a high free sugar content, such as soft drinks were high (114.4 ± 6.7 g) and to a lesser extent sweets (40.2 ± 0.9 g).

Overall mean average daily energy intake was around 2600 kcal, which represented 104.1 [103.1, 105.0]% of requirements (Table 2). The mean percentage contributions to total energy intake of carbohydrates were 56.2[55.8, 56.7]%, protein 13.6[13.5, 13.7]% and fat 28.1[27.7, 28.5]%.

**Table 2:** Macro and micronutrients intakes /1000 kcal, overall and by gender, 20–49 y. in Greater Tunis

	All (*n* = 2545)		Women (*n* = 1651)		Men (*n* = 894)		Women vs. Men		
	Mean^a^	SEM^b^	Mean^a^	SEM^b^	Mean^a^	SEM^b^	Diff.^c^	95% CI^d^	P^e^
Energy									
Energy intake (kcal)	2578.6	15.1	2300.4	12.6	2858.8	32.28	− 558.4	− 607.2, − 509.5	<0.0001
Energy intake (kJ)	10,789.0	63.2	9624.9	52.6	11,961.1	97.4	− 2336.2	− 2540.6, − 2131.7	<0.0001
Energy intake as % of requirements	104.1%	0.5	105.5%	0.5	102.6%	0.7	+2.9%	1.3, 4.5	0.001
Macronutrients (/1000 kcal)									
Protein (g)	34.0	0.2	33.7	0.1	34.2	0.2	−0.4	− 0.9, 0.1	0.10
Carbohydrate (g)	140.5	0.5	140.8	0.6	140.0	0.6	+ 0.8	−0.5, 2.1	0.22
Free sugar (g)	31.3	0.4	31.9	0.5	30.8	0.7	+ 1.1	−0.4, 2.5	0.15
Dietary fiber (g)	10.9	0.1	10.8	0.1	10.9	0.1	−0.1	−0.3, 0.1	0.36
Total fat (g)	31.2	0.2	31.1	0.2	31.3	0.2	−0.2	−0.6, 0.3	0.56
Saturated fat (g)	7.50	0.1	7.6	0.1	7.4	0.1	+ 0.2	−0.1, 0.4	0.15
Monounsaturated fat (g)	12.0	0.1	12.1	0.1	11.9	0.1	+ 0.2	−0.1, 0.4	0.12
Polyunsaturated fat (g)	8.9	0.1	8.7	0.1	9.0	0.1	−0.3	−0.5, − 0.1	0.0009
Omega-3 fat (mg)	260.8	4.4	248.8	5.3	272.8	6.4	−24.0	−39.6, −8.4	0.0029
Omega-6 fat (mg)	2653.6	34.2	2654.5	41.1	2652.7	49.6	+ 1.8	− 118.3, 121.9	0.98
Micronutrients (/1000 kcal)									
Cholesterol (mg)	84.8	1.6	82.5	1.8	87.3	2.0	−4.8	−9.0, −0.6	0.026
Calcium (mg)	276.1	2.6	288.0	2.9	264.2	3.5	+ 23.8	16.6, 31.0	<0.0001
Iodine (mg)	82.1	1.3	81.9	2.1	82.3	1.2	−0.4	−4.6, 3.9	0.86
Iron (mg)	7.4	0.1	7.3	0.1	7.4	0.1	−0.2	−0.3, − 0.1	0.012
Magnesium (mg)	116.4	0.7	116.5	0.8	116.3	1.0	+ 0.2	−1.6, 2.0	0.79
Sodium (mg)	1498.2	11.7	1496.5	17.8	1499.8	13.7	−3.3	−45.9, 39.4	0.88
Phosphorus (mg)	470.7	3.2	479.1	2.0	462.3	5.6	+ 16.8	6.9, 26.8	0.0012
Potassium (mg)	1032.5	7.0	1044.7	7.5	1020.3	9.9	+ 24.4	3.1, 45.8	0.025
Vitamin C (mg)	141.9	2.0	129.2	2.0	154.8	3.5	−25.6	−33.4, −17.8	<0.0001
Zinc (mg)	3.8	0.1	3.7	0.1	3.8	0.1	− 0.1	−0.1, 0.1	0.38

^a^Weighted mean (accounting for unequal probabilities of selection and differential response rates)

^b^SEM: standard error of the mean taking into account sampling design

^c^Women vs. Men difference of means (unadjusted)

^d^95% confidence interval taking into account sampling design

^e^*P* value for Women vs. Men difference

Overall, mean DQI-I was 59.5 ± 0.3 with a minimum of 34.6 and maximum of 79.5 and 50.4 [47.2, 53.7]% of the subjects had a good diet quality (Table 3). The overall adequacy score was the highest at 79.1% of the 40 maximum possible score while variety (55.5% of 20) and moderation (44.2% of 30) were at much lower levels and balance the lowest (34.6% of 10). Within component scores varied somewhat, e.g. in the adequacy category, most items were close to their 5.0 maximum score, e.g. grain, fibre, protein, iron and vitamin C, in accordance with food and nutrient data. On the other hand, fruits and vegetables scores were lower and mild, respectively (38.4 and 54.1% of maximum score). In the moderation component, there were high sub-scores for saturated fat and cholesterol (each about 80% of 6) but a mean score of only 2.1 (over a maximum of 6) for the total fat item and a very low score (less than a third of the maximum achievable) for sodium. The lowest of all scores was for the empty calories items in the moderation component (0.1 over 6, i.e. about 2% of the maximum score).The fatty acids ratio in the balance sub-component was also quite low at 0.8 (over 5).

**Table 3:** Diet Quality Index-International and sub-scores overall and by gender, 20–49 y. in Greater Tunis

	All (*n* = 2545)		Women (*n* = 1651)		Men (*n* = 894)		Women vs. Men		
	Mean^a^	SEM^b^	Mean^a^	SEM^b^	Mean^a^	SEM^b^	Diff.^c^	95% CI^d^	P^e^
Diet Quality Index-International (/100)	59.5	0.3	58.6	0.3	60.4	0.3	− 1.8	− 2.6, − 1.0	<0.0001
Variety score (/20)	11.1	0.1	10.1	0.1	12.1	0.2	−2.0	− 2.3, − 1.6	<0.0001
Overall food group variety	9.4	0.1	8.8	0.1	10.0	0.1	−1.3	− 1.5, − 1.0	<0.0001
Within-group variety for protein source	1.7	0.0	1.4	0.1	2.1	0.1	−0.7	− 0.9, − 0.6	<0.0001
Adequacy score (/40)	31.6	0.1	30.8	0.1	32.5	0.1	−1.8	−2.0, − 1.5	<0.0001
Grain group	4.7	0.0	4.7	0.1	4.7	0.1	0.0	−0.1, 0.1	0.44
Fruit group	1.9	0.1	2.1	0.1	1.8	0.1	+ 0.3	0.2, 0.5	0.0003
Vegetable group	2.7	0.0	2.7	0.1	2.7	0.1	+ 0.0	−0.1, 0.1	0.85
Dietary fibers	4.8	0.0	4.7	0.1	4.8	0.1	−0.1	−0.1, 0.0	0.0032
Protein	5.0	0.0	5.0	0.1	5.0	0.1	+ 0.0	−0.1, 0.1	0.29
Iron	4.2	0.0	3.4	0.1	5.0	0.1	−1.6	− 1.7, − 1.6	<0.0001
Calcium	3.4	0.0	3.2	0.1	3.6	0.1	−0.4	−0.5, − 0.3	<0.0001
Vitamin C	5.0	0.0	5.0	0.1	5.0	0.1	+ 0.0	−0.1, 0.1	0.73
Moderation score (/30)	13.2	0.1	14.2	0.2	12.3	0.2	+ 1.8	1.4, 2.2	<0.0001
Total fat	2.1	0.1	2.2	0.1	2.1	0.1	+ 0.1	−0.1, 0.2	0.39
Saturated fat	4.6	0.1	4.5	0.1	4.6	0.1	−0.1	−0.3, 0.1	0.28
Cholesterol	5.0	0.1	5.4	0.1	4.7	0.1	+ 0.7	0.5, 0.9	<0.0001
Sodium	1.4	0.1	1.9	0.1	0.8	0.1	+ 1.2	1.0, 1.3	<0.0001
Empty calorie foods	0.1	0.1	0.1	0.1	0.1	0.1	+ 0.0	−0.1, 0.1	0.42
Overall balance score (/10)	3.5	0.1	3.5	0.1	3.4	0.1	+ 0.1	−0.2, 0.4	0.42
Macronutrient ratio	2.6	0.1	2.7	0.1	2.6	0.1	+ 0.1	−0.1, 0.3	0.58
Fatty acid ratio	0.8	0.0	0.9	0.1	0.8	0.1	+ 0.0	−0.1, 0.2	0.46

^**a**^Weighted mean (accounting for unequal probabilities of selection and differential response rates)

^**b**^SEM: standard error of the mean taking into account sampling design

^**c**^Women *vs.* Men difference of means (unadjusted)

^**d**^95% confidence interval taking into account sampling design

^**e**^*P* value for Women *vs.* Men difference. Diet quality as assessed by the DQ-I (Diet Quality Index-International) and sub-components (variety, adequacy, moderation and balance), derived from the 3-day prospective food record, overall and by gender, among 20-49 year old subjects in the Greater Tunis area, Tunisia

### Gender differences in dietary intake and diet quality

There were no huge differences between women and men in their daily average consumption of the food groups when expressed in g/1000 kcal (Fig. 1). Nevertheless, women consumed more fruit than men (+ 11.5 [3.2, 19.9] g, *P* = 0.0074). They consumed less red meat than men (− 2.7 [− 4.3, − 1.1] g, *P* = 0.001), but there was no difference in the consumption of white meat, fish, eggs and dairy products. Women’s intakes of sweets were also higher (+ 2.9 [1.5, 4.3] g, *P* < 0.0001) but they consumed much less soft drinks (− 13.8 [− 19.9, -7.7] g, *P* < 0.0001). Women consumed somewhat more seed oil than men (+ 0.6 [0.2, 1.0] g, *P* = 0.0041) but less olive oil (− 0.9, [− 1.4, -0.5] g, *P* < 0.0001).

Although mean average absolute daily energy intake was much lower for women than men, as a % of recommendation levels, it was slightly higher for women. Concerning macronutrients expressed as /1000 kcal (Table 2) women reported lower intake of omega-3 and a higher micronutrient intake of calcium and phosphorus but lower intake of vitamin C. Iron intakes were similar for women and men, but when expressed as % of recommendations, much lower for women: 51.6%, vs. 100.0% for men.

**Fig. 2:**
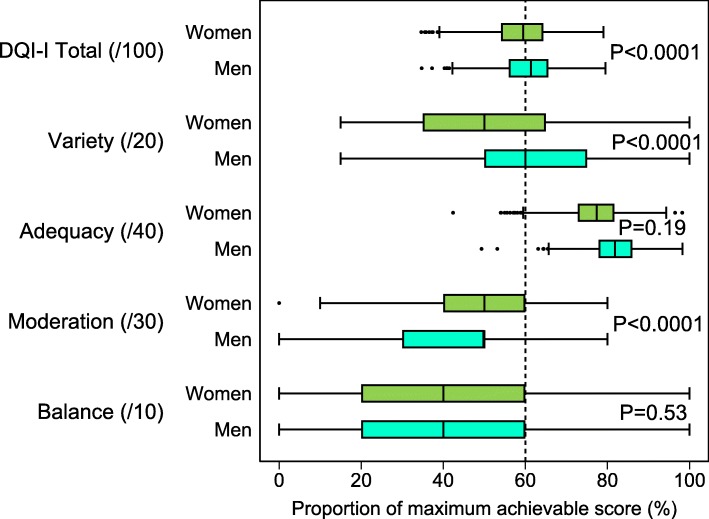
Distribution of DQI-I and sub-scores, by gender, among 20–49 y. in Greater Tunis. DQI-I (Diet Quality Index International) and sub-components scaled as proportion of the maximum achievable score (women n = 1651, men n = 894). Box-plots: box is weighted interquartile range, the vertical bar inside the box is the weighted median, whiskers extend 1.5 interquartile range on each side of the box, values outside the whiskers are plotted individually. *P*-Value: chi-square test (taking into account sampling design) for the gender contrast in the proportion of subjects with > 60% of maximum achievable score (vertical dotted line)

Women had a lower overall DQI-I score than men, and also the proportion of subjects with a good diet quality (DQI-I > 60) was lower among women than men women and men (Table 3, Fig. 2). However, gender differences varied with the four sub-components: women had lower mean variety and adequacy scores (for the latter, women scored a little better than men for fruit, but had much lower scores than men for iron and somewhat lower scores for calcium). Conversely, women had better moderation scores than men, mostly due to their better score on the cholesterol and sodium items. There was no difference between men and women in the balance subcomponent.

### Socio-demographic patterning of gender differences in diet quality

There was a reduction in differences between women and men in DQI-I, with age whether crude or adjusted. In unadjusted analyses, DQI-I gender differences increased with the economic level of the household (but this did not persist in adjusted analyses). Adjusted gender differences were higher in larger than in smaller households. These differences detrimental to women, were more marked among subjects in the extreme categories of education i.e. in the no-schooling and university categories vs. no significant differences in the middle categories. Mostly similar trends were observed in the gender odds ratio for DQI-I > 60, although not always significant. On the contrary, the gender contrast in diet quality was stronger in urban than in rural settings only for the DQI-I > 60 binary variable with the same but not significant trend for the interval variable (Table 4).

**Table 4:** Diet quality gender inequalities by geographic and socioeconomic variables, 29–40 y. in Greater Tunis

			Diet Quality Index-International (DQI-I)								Diet Quality Index-International (DQI-I) > 60					
	Women	Men	Women		Men		Women vs. men				Women	Men	Women vs. men			
	*n*	*n*	Mean^a^	SEM^b^	Mean^a^	SEM^b^	Crude Diff.^c^	95% CI^d^	Adjusted Diff.^e^	95% CI^d^	%^f^	%^f^	Crude OR^g^	95% CI^h^	Adjusted OR^i^	95% CI^h^
Area							*P*^**j**^ = 0.25		P^**j**^ = 0.30				*P*^**j**^ = 0.17		P^**j**^ = 0.079	
Urban	1437	774	58.5	0.3	60.4	0.4	−1.9	−2.7, − 1.0	−1.7	−2.9, −0.6	44.2	55.3	0.6	0.5, 0.8	0.7	0.5, 0.9
Rural	214	120	59.5	0.5	60.4	0.7	−1.0	−2.3, 0.3	−0.8	−2.6, 1.0	57.0	60.0	0.9	0.6, 1.4	1.0	0.6, 1.7
Governorates							*P*^**j**^ = 0.81		P^**j**^ = 0.61				*P*^**j**^ = 0.76		P^**j**^ = 0.66	
Tunis	680	360	58.8	0.4	60.1	0.6	−1.3	−2.6, −0.1	− 1.0	−2.4, 0.5	44.7	52.4	0.7	0.5, 1.0	0.8	0.6, 1.2
Ariana	365	208	57.6	0.9	59.8	0.6	−2.2	−4.3, −0.1	− 2.1	− 4.3, 0.0	41.4	56.1	0.6	0.4, 0.8	0.6	0.4, 1.0
Ben Arous	343	210	58.7	0.6	60.7	0.6	−2.0	−3.5, −0.5	−1.9	−3.4, −0.5	46.3	58.1	0.6	0.4, 0.9	0.6	0.4, 1.0
Manouba	263	116	59.2	0.7	61.4	0.8	−2.2	−4.1, −0.3	− 2.3	−4.5, −0.1	50.0	60.3	0.7	0.4, 1.0	0.7	0.4, 1.0
Age (years)							*P* ^**j**^ = 0.048		P^**j**^ = 0.11				*P*^**j**^ = 0.18		P^**j**^ = 0.51	
20–29	497	248	56.4	0.5	59.3	0.6	−2.9	−4.5, −1.3	− 2.2	−4.2, −0.2	35.1	51.3	0.5	0.4, 0.7	0.6	0.4, 1.1
30–39	486	261	59.4	0.4	61.1	0.5	−1.6	−2.6, −0.6	−2.0	−3.4, −0.6	50.7	58.5	0.7	0.6, 0.9	0.7	0.5, 1.0
40–49	668	385	60.5	0.3	61.1	0.4	−0.6	−1.7, 0.4	−0.4	−2.0, 1.2	52.7	59.0	0.8	0.6, 1.0	0.9	0.6, 1.3
Marital status							*P*^**j**^ = 0.058		P^**j**^ = 0.98				*P*^**j**^ = 0.066		P^**j**^ = 0.74	
Married	1057	607	59.9	0.3	61.1	0.3	−1.3	−2.0, −0.5	− 1.7	−3.2, − 0.1	52.1	59.3	0.7	0.6, 0.9	0.7	0.5, 1.1
Other	594	287	56.5	0.5	59.3	0.6	−2.8	−4.3, −1.3	−1.7	−3.2, −0.2	34.6	50.9	0.5	0.4, 0.7	0.7	0.4, 1.0
Household size							*P*^**j**^ = 0.30		P^**j**^ = 0.13				*P*^**j**^ = 0.39		P^**j**^ = 0.28	
1–3	234	147	59.5	0.4	61.2	0.7	−1.7	−2.9, −0.4	− 1.0	−2.4, 0.4	49.8	57.9	0.7	0.5, 1.0	0.9	0.6, 1.3
4–5	900	508	59.2	0.3	60.5	0.4	−1.3	−2.1, −0.5	− 1.1	−2.1, − 0.1	48.2	56.1	0.7	0.6, 0.9	0.8	0.6, 1.3
6 or more	517	239	57.6	0.5	60.0	0.6	−2.4	−3.9, −0.9	− 2.4	−4.1, − 0.8	40.6	54.7	0.6	0.4, 0.8	0.6	0.4, 0.9
Education							*P*^**j**^ = 0.0006		P^**j**^ = 0.12				*P*^**j**^ = 0.0042		P^**j**^ = 0.16	
No formal schooling	179	40	60.1	0.6	63.0	1.4	−2.9	−5.8, −0.1	−3.3	−6.4, − 0.2	52.1	78.0	0.3	0.1, 0.9	0.3	0.1, 0.9
Primary school	601	316	59.8	0.4	60.4	0.5	−0.5	−1.8, 0.7	−0.9	−2.5, 0.8	52.0	57.4	0.8	0.6, 1.1	0.8	0.5, 1.2
Secondary	567	377	58.0	0.4	59.4	0.5	−1.4	−2.6, −0.2	−1.1	−2.4, 0.3	42.2	48.9	0.8	0.6, 1.0	0.9	0.6, 1.2
University	304	161	56.9	0.7	62.5	0.8	−5.6	−7.5, −3.7	−3.7	−5.9, −1.5	36.9	66.6	0.3	0.2, 0.5	0.5	0.3, 0.9
Professional activity							*P*^**j**^ = 0.26		P^**j**^ = 0.90				*P*^**j**^ = 0.11		P^**j**^ = 0.80	
Upper/intermediate	169	264	58.4	0.9	61.0	0.6	−2.6	−4.5, −0.7	−1.0	−2.9, 0.9	42.5	56.3	0.6	0.3, 1.0	0.7	0.4, 1.3
Employee/worker	374	504	57.9	0.5	60.1	0.4	−2.2	−3.7, −0.7	−1.9	−3.3, − 0.5	44.6	56.3	0.6	0.4, 0.9	0.7	0.4, 1.0
Not working/retired	934	61	59.6	0.3	60.3	0.9	−0.7	−2.7, 1.3	−1.7	−3.8, 0.3	49.9	47.6	1.1	0.6, 1.9	0.9	0.5, 1.6
Student	174	65	55.9	0.8	60.0	1.4	−4.1	−7.3, −0.9	−1.8	−5.4, 1.8	30.3	56.3	0.3	0.2, 0.7	0.5	0.2, 1.3
Household welfare proxy							*P*^**j**^ = 0.12		P^**j**^ = 0.47				*P*^**j**^ = 0.31		P^**j**^ = 0.68	
Lower tertile	566	288	58.8	0.4	60.1	0.4	−1.3	−2.5, −0.2	−1.5	−3.0, −0.1	47.0	55.3	0.7	0.5, 1.0	0.7	0.5, 1.0
Intermediate tertile	542	312	59.1	0.4	60.2	0.6	−1.2	−2.5, 0.2	−1.2	−3.0, 0.6	48.5	55.7	0.8	0.5, 1.1	0.8	0.5, 1.3
Upper tertile	543	294	57.8	0.5	60.7	0.6	−2.9	−4.3, −1.5	− 2.3	−3.7, −0.8	39.9	56.1	0.5	0.4, 0.8	0.6	0.4, 0.9

^a^Crude weighted mean (accounting for unequal probabilities of selection and differential response rates)

^b^SEM: standard error of the mean taking into account sampling design

^c^Crude women vs. men difference of means within category of co-variable as in column 1

^d^Diff. 95% confidence interval taking into account sampling design

^e^Adjusted women vs. men difference of means within category of socio-demographic variable: multivariate model including all variables in first column, energy intake and interactions with gender

^f^Crude weighted prevalence (accounting for unequal probabilities of selection and differential response rates)

^g^Crude women vs. men (DQI-I > 60) prevalence odds-ratio (OR) within category of covariable as in column 1

^h^OR 95% confidence interval taking into account sampling design

^i^Adjusted women vs. men (DQI-I > 60) prevalence odds-ratio (OR) within category of socio-demographic variable: multivariate model including all variables in first column, energy intake and interactions with gender

^j^Crude or adjusted *P* value for gender x variable interaction: null hypothesis of identical gender contrasts (difference of means or OR) in all categories of socio-demographic variable

## Discussion

Our study compared dietary intakes between women and men in a nutrition transition context where the prevalence of excess adiposity is overall high and there are strong gender inequalities detrimental to women in both excess adiposity and anaemia [15]. Overall diet quality was average in our population as only half the subjects had a good quality diet according to the DQI-I. We found mild gender differences in terms of food groups as well as energy or micro-nutrients. As assessed by the DQI-I, diet quality was somewhat lower for women than men (but women had a better moderation sub-score than men). The patterning of these diet quality gender inequalities according to socio-demographic characteristics was not marked, although these inequalities varied somewhat with age, household size and education.

### Energy intake, overall and by gender

Energy intake was high, all the more as the 3-day food record methods is known to be prone to under reporting bias [20]. It was lower than reported in a survey in Greater Tunis in 2006 but the subjects were somewhat older and the survey used a food frequency questionnaire (known to be more prone to over reporting than the 3-day record) [41]. Energy intake was nevertheless comparable to values observed in the same age-category e.g. in Lebanon [42]. Energy intake was also much higher than observed e.g. in France at the same period, in line with the nutrition transition that Tunisia is experiencing, all the more in urban contexts [12, 43]. Energy intake as % of requirements was only slightly higher for women. This could be considered as somewhat unexpected given the marked gender overweight/obesity inequalities detrimental to women in this context, and also with hypotheses that these inequalities could be linked to differential gender roles which would foster higher dietary intake among women vs. men [15, 44]. But cross-sectional studies have a number of known limitations for the study of associations between diet and health outcomes.

### Food groups and nutrients intake overall and by gender

Our results revealed high consumption of white bread, even higher than among Tunisian adolescents, more than twice that observed in France, for example, and also much higher than in another urban setting in the MENA region in Lebanon [35, 42, 45]. Also in the cereal group, consumption of pasta was especially high, in line with worldwide data on pasta production and imports which shows that Tunisians are the second highest consumers of pasta /inhabitant/year worldwide [46]. Consumption of animal products was still moderate compared to developed countries or even Lebanon [45]. Consumption of fruit and vegetables in absolute values (431.1 (± 9.5) g/d) was somewhat higher than that observed in Beirut or in France [42, 45]. Soft drinks were among the top five most consumed food groups, even more than observed for adolescents in the same context [35, 47]. The overall structure of food consumption was in line with trends towards an increase in consumption of “modern” foods in the context of the nutrition transition, though consumption of Mediterranean diet staples such as fruits and vegetables was still sizeable.

Women consumed (for 1000 kcal/d) more fruit than men and less soft drinks consistent with the results of several previous studies [48–50]. Women also consumed more sweets than men and less red meat, consistent with studies showing that men prefer hot hearty food such as steak, while women prefer snacks (such as chocolate and ice cream) [51]. Also sweets have been shown to be culturally associated with femininity [52] while meat is commonly associated with masculinity [53]. This difference could stem both from actual gender differences in dietary intake regarding these “feminine” vs. “masculine” foods or reporting bias linked to social desirability issues or a combination of both [54]. There was no gender difference in total fat intake but women consumed less olive oil and more seed oil than men, which could explain lower intakes of polyunsaturated fat by women than by men. There were no gender differences in the consumption of other macronutrients. Concerning micronutrients, women had higher intakes /1000 kcal of phosphorus, potassium and calcium than men, but women had lower vitamin C intake and also marginally lower iron intake. Higher recommended iron intakes for women, combined with possibly reduced bioavailability of iron due differential calcium and vitamin C intake, could be an issue in relation with the gender differential regarding anaemia and/or iron deficiency observed in this context [15, 17]. Regarding the consumption of more “modern” vs. more “traditional” foods as well as nutrients, in this nutrition transition context the observed gender differences were thus not uni-directional.

### Overall diet quality

Concerning the sub-components of the global DQI-I score, mean variety only reached half the maximum score, less than previous studies in Tunisia [35, 41], but these surveys were of different age groups and/or in different areas. Also in our study variety was notably lower than that reported in the USA two decades earlier [33]. The variety of protein sources item scored especially low, partly due to low fish consumption (only 4.7% consumed at least 0.5 of a serving). The adequacy component scored the highest (around 80% of the maximum score), comparable to that reported in other studies in the same context [35, 41] and higher than that observed in southern Spain [55] or even South Korea, China and the USA [33, 56]. Scores on items such as grain, fibres, protein, iron and vitamin C were particularly high, in relation with intakes of the food groups reported in Fig. 1. Scores for the fruits and vegetables items were the lowest: indeed the cumulated consumption of these two food groups, although more than that recorded among women in Rabat (Morocco), was only slightly above the recommended 400 g/d [31, 57]. The score for calcium was only average due to a consumption of dairy products of less than 100 g/d, still relatively low compared to that observed in France, or even Lebanon [42, 45]. Indeed, apart from a few products such as “leben” (fermented milk), dairy products were never a main constituent of the traditional dietary intake in the context [58]. But in Tunisia a “modern” dietary pattern was shown to be correlated with increased consumption of dairy products among adolescents [35, 59]. Moderation (44% of the maximum score) was rather low compared to that reported in most of the studies cited above. Remarkable is the almost null score on the empty calorie foods item, including soft drinks (indicating high consumption) and the low scores on the total fat and sodium items in line with the nutrition transition that Tunisia is experiencing [12, 35]. The mean absolute sodium intake (3847.5 (± 33.4) mg) was about twice the WHO recommended threshold [31]. It is nevertheless encouraging that the mean score for saturated fat was rather high at 4.6 (out of a maximum of 6), likely related to the still moderate consumption of animal products, compared to that in developed countries or even in related contexts [42, 45]. The lowest proportion of maximum score was obtained for the balance sub-component (34.6%): it was nevertheless higher than that observed in the other studies which used DQI-I cited above [33, 56, 60].

As a result, mean DQI-I was about 60 while only half the subjects had a DQI-I > 60, comparable to e.g. mean value of 60.4 (± 0.4) in Greater Tunis [41]. The national study of 15–19 years adolescents in Tunisia in 2005 showed a lower mean DQI-I (57.7 (± 0.3)) along with a much lower proportion of DQI-I > 60 (38%) [35]. Conversely, a study on Tunisian men (though from a specific sample matched to Tunisian migrants in France) revealed a quite high overall mean DQI-I of 64.5 (± 0.6) [60]. Comparable data in other countries in the MENA region are scarce, but a study in southern Spain (ie, the north Mediterranean area) reported a much lower diet quality (mean DQI-I of a 46.3) although the survey focussed on a different age category (6–18 year old subjects) [55]. The diet quality in our population was comparable to that observed in two large scale surveys in China and the USA [33] though two decades earlier (consistent with the nutrition transition having started in the context of our study only a few decades ago).

### Gender differences in diet quality

Women had less variety in their diet than men: in some contexts discrimination in intra-household food allocation unfavourable to women has been documented, but there is no such data in our context [61]. Adequacy was quite lower for women vs. men mostly due to a lower score on the iron intake item, as was found also in Korea [56], despite a similar absolute iron intake between both. This is consistent with a large anaemia gap to the detriment of women found in the same population, about half of which was due to iron deficiency [15]. As is the case of many observed women vs. men inequalities, the lower iron score may originate from both from sex-linked biological differences, as women of childbearing age have higher iron requirements, but also from the social context. Indeed, if gender equality pertains only to absence of gender differences, diet equity should focus on women and men’s needs whether similar or different [5]. Beyond the absence of gender based negative discrimination regarding micronutrient-rich food observed in our study (but which has sometimes been observed in some contexts), this would require positive discrimination regarding that aspect of the diet to account for women’s specific iron requirements, for example [61]. Women also had a marginally lower adequacy score for calcium (despite higher intakes in 1000 kcal/d), also due to their specific requirements. On the other hand they also had a slightly higher score for fruit, consistent with food group data, which has also been reported in other studies [62, 63]. In a context of a low overall moderation score, women scored somewhat better than men on this component which assesses intake of food and nutrients related to NCDs and may need to be restricted [33]. This was mostly due to women’s better scores for cholesterol and sodium, consistent with worldwide data [13]. On the other hand, both women and men had extremely low scores for empty calorie foods (which includes soft drinks and sweets). In our context, where gender roles are even more marked than in the context in which the other studies were conducted [64, 65] gender stereotypes regarding food choice (that we discussed above) could be a possible pathway to explain this better moderation score for women. Both women and men had a poor overall balance score, as also found in Korea and among Tunisian adolescents [35, 56].

Overall, in our context, the mean DQI-I as well as the proportion of good quality diet (DQI-I > 60) was lower among women. In general, men give less importance to healthy eating than women [66, 67], which has been observed in several countries [63]. Also a worldwide meta-analysis underlined the fact that women scored better than men both on greater consumption of healthy dietary items and of less consumption of unhealthy dietary items [13]. On the other hand, a study conducted in Korea showed no difference between women and men [56]. Comparable data in analogous contexts are scarce, e.g. no gender difference was found in Morocco but different instruments were used both for assessing dietary intake and for scoring diet quality [68]. Beyond overall lower quality, the women in our study had better adequacy scores for fruit as well as better moderation scores for sodium and cholesterol. This may be one of the pathways by which they are protected against cardiovascular diseases, despite their much higher excess adiposity in our context [69–71]. Concerning energy intake relative to requirements, observed gender differences in diet quality were generally not huge in comparison with the marked inequalities in excess adiposity in our setting. Beyond the factors discussed above concerning energy, some authors reported a stronger link between diet quality and obesity among women than men [72]. So that the same observed difference between women and men regarding diet quality, could have more impact on the contrasts between women and men in excess adiposity than the same observed difference e.g. between two different populations of men (e.g. from different areas of residence or milieus). As mentioned above, further research is needed on this issue, du to the limitation of cross-sectional studies.

Overall, these results point to ways to explore for gender specific prevention as we showed that gender contrasts depend on which dimension of diet quality is considered.

### Socio-demographic patterning of gender differences in diet quality

The difference decreased with age, and were null in the 40–49 age category: the decrease was also observed for obesity and is consistent with documented diminishing gender role differentials with age [9, 10, 15]. Interestingly, we showed that gender inequalities in DQI-I (either as intervals or as binary variables) detrimental to women were worse in the extreme categories of education. On one hand, women with no schooling who are less likely to work outside the home and/or in an environment with more inegalitarian household roles are mostly in charge of preparing meals and would thus receive more food stimuli than men, including unhealthy foods [9, 73]. On the other hand, a previous study in our context showed that women with a higher level of education significantly favoured somewhat slimmer silhouettes [74]. This could lead to the adoption of a lower quality diet due to intake restrictions vs. men whose body image is less socially constrained [75]. Contrasted social disadvantage issues in the no formal schooling vs. superior categories would result in similar gender differences in diet, higher than for the intermediate categories (as a sort of a “non-linear” intersectionality). Also, the gender difference in diet quality was more important in large households than in medium or smaller ones. This is possibly due to higher gender differentials in intra-household food allocation or to the other pathways discussed above, both linked with more inegalitarian gender roles in these probably more traditional larger households. Concerning area of residence, the gender contrast in diet quality was more detrimental to women in urban areas than in rural areas (at least using the DQI-I > 60 binary indicator, while the same trend was observed for the DQI-I interval variable, it was not statistically significant). This may seem paradoxical as gender differences in socio-economic indicators are less marked in urban than in rural areas, so resulting in less inegalitarian gender roles in urban areas. But we need to underline the observed non-linear association of gender inequalities with socio-economic indicators, e.g. education or household size so that the latter interpretation may not be as straightforward as it would seem. Also, as discussed in the case of obesity [10], it could be that variations in gender inequality in the diet as a function of socioeconomic factors depends on the level of aggregation at which the association is assessed (difference between contextual and individual or household level, which underlies multilevel analyses [76]). But overall, the small sample size for rural participants (reflecting the mostly urban study area), is a notable limitation to these urban vs. rural comparisons.

### Strengths and limitations of the study

The study area is the most urbanized and developed region of Tunisia, as a case study of an “advanced nutrition transition” setting where gender issues are of concern. The generalizability would then pertain more to similar urban settings in the MENA region than Tunisia as a whole (where 30% of the population is rural). There was a quite lower response rate for men vs. women (not unusual in the context) which was only partially taken into account by post-stratification weights. Although the distribution of socio-demographic variables among men does not suggest a major selection bias, it cannot be ruled out that such a bias may have influenced our diet gender inequality estimates. Nevertheless a strength of our study was to compare dietary intake of women and men from a large random sample. Cross sectional studies have limitations regarding the assessment of associations but our main exposure (gender) is likely not prone to reverse-causality bias. All dietary assessment instruments (e.g. our 3-day food record method) are prone to measurement biases [20]. Also like for all food consumption measurements, the reporting of intake of each food group may be biased to a different degree due to social desirability or approval and the bias could be differential e.g. depending on gender or weight status [54, 77]. Theoretically, infection, or enteropathy or inflammation may result in increased energy requirements, reduced energy intake or even increased nutritional losses and could impact the interpretation of measured dietary intake. Nevertheless, our study area is characteristic of a developed environment (also in a mostly urban area) in an advanced epidemiological transition situation, where infections/entheropathies are residual. Low grade inflammation due to a high prevalence of excess adiposity has been documented in an analogous context [78] but was not taken into account in the interpretation of dietary intake (as in most similar studies pertaining to food consumption). Other a priori scores of diet quality other than the DQI-I could have been used [79]. But using DQI-I enabled us to assess different dimensions of diet quality, especially in our nutrition transition context, the moderation component (for which we found a significant difference in favour of women). We could also have analysed gender differences in diet through a posteriori data driven multivariate dietary patterns [80] (a possible direction for future research). Another strength of our study was the quantitative assessment of women vs. men diet using relevant gender inequality measures. We also studied their variation with socio-demographic factors, by analogy with the concept of intersectional analysis in qualitative research, to understand the possible differential association of gender with diet in different socioeconomic conditions [5].

## Conclusions

Overall, in a typical nutrition transition context in the MENA region, both women and men had relatively high scores for “healthy items” (e.g. in the adequacy component) while the components pertaining mostly to “unhealthy items” were scored lower (e.g. in moderation and balance). This is in line with worldwide trends towards increases in consumption of both “healthy” and “unhealthy” food items in middle income countries [13]. As the women’s higher moderation score than that of men did not make up for their lower variety and adequacy scores, overall diet quality was somewhat lower for women than men, contrary to that observed in other contexts. Observed gender differences in dietary intake were nevertheless mild. These differences in diet quality varied somewhat according to sociodemographic indicators linked to different gender roles.

Our results nevertheless suggest possible ways to explore for gender specific prevention, as we showed that gender contrasts depend on which dimension of diet quality is considered (e.g. better moderation but worse adequacy and variety for women). Beyond equality, prevention should aim at gender equity (which implies that women are subject to positively discrimination to account for their specific requirements). Generally, in this context where gender issues are substantial and socio-culturally deep rooted, the reduction of women vs. men differences, including in health and nutrition is likely a long term process.

## Additional files

Additional file 1: **Table S1***.* Food list (203 items) and food groups (20) consumed by 20–49 year old subjects in Greater Tunis. List of the 203 food items collected from the 3-day food record and list of the 20 food groups derived from the food items. (DOCX 14 kb)

## Abbreviations

DQI-I: Diet Quality Index International
FAO: Food and Agriculture Organization of the United Nations
MENA: Middle East and North Africa
NCD: Non-Communicable Diseases
WHO: World Health Organization
