# Supplementary material for: Gender inequalities in diet quality and their socioeconomic patterning in a nutrition transition context in the Middle East and North Africa: a cross-sectional study in Tunisia
Source: Nutr J. 2019 Mar 21;18:18. doi: 10.1186/s12937-019-0442-6 (PMC6427851; doi:10.1186/s12937-019-0442-6)
Supplement: Supplementary file 1 — Table S1. Food list (203 items) and food groups (20) consumed by 20–49 year old subjects in Greater Tunis. List of the 203 food items collected from the 3-day food record and list of the 20 food groups derived from the food items. (DOCX 14 kb) [file 12937_2019_442_MOESM1_ESM.docx]

**Table S1**

Food list (203 items) and food groups (20) consumed by 20-49 y., Greater Tunis.

| **Food groups** | **Food items from prospective 3-day food record** |
| --- | --- |
| 1-White bread | french bread |
| 2-Other cereals | corn starch, corn, dry whole grain wheat, cracked wheat, corn flakes, couscous, pearled barley, |
|  | sorghum flour, flour dough, semolina, pasta, rice, unsalted bread, barley bread, corn bread, |
|  | soybean oil bread (*mbasses*), traditional bread (*mlawi*), country style bread (*tabouna*), |
|  | homemade bread (*tagine*), unsalted rusk bread, rusk bread, whole meal bread, loaf bread |
| 3-Fruits | apple, strawberry, pomegranate, lemon, kiwi, melon, blackberry, medlar, orange, pear, prickly pear, |
|  | watermelon, apricot, banana, cherry, clementine, quince, jam, dates, fig, peach, plum, grape |
| 4-Vegetables | cucumber, artichoke, eggplant, beets, swiss chard, cardoon, carrot, celery, green pepper, tomato, |
|  | cabbage green, cauliflower, coriander, pumpkin, zucchini, spinach, fennel bulb, turnip, leek, |
|  | fennel leaf, mushroom, parsley, green pea, green pepper, red pepper powder, popcorn, radish |
|  | fenugreek, okra, jute corchorus olitorius (*mloukhia*), diplotaxis hara (*hara*), green bean, lettuce |
| 5-Soft drinks | energy drink, light soda, soda, syrup |
| 6-Dairy products | melted cheese, semi-hard cheese, semi-skimmed milk, condensed & sweetened milk, |
|  | skimmed milk, whole milk, leben**^a^**, little swiss cheese, ricotta, natural yogurt, semi-skimmed yogurt |
| 7-Water/tea/coffee | coffee, prepared coffee, water, nescafe, tea |
| 8-Potatoes | potato crisps, french fries, potatoes |
| 9-Sweets | chocolate biscuit, biscuit, cake, chamia**^b^**, black chocolate bar, milk chocolate bar, chocolate |
|  | powder, hazelnut chocolate spread, chocolate cream, fresh cream, flan, ice cream, honey, |
|  | citron sorbet, sugar, sweets |
| 10-White meat | chicken liver, turkey, turkey liver, chicken |
| 11-Red meat | beef liver, beef kidney, beef tripe, beef meat, beef ground meat, camel meat, goat meat, |
|  | lamb intestine, lamb heart, lamb liver, lamb fat, lamb lung, lamb kidney, lamb meat hamburger, |
|  | sheep meat, sheep ground meat, rabbit |
| 12-Legumes | broad bean, kidney bean, lentils, chickpeas |
| 13-Eggs | whole egg, egg yolk, egg white |
| 14-Seed oil | corn oil, soybean oil |
| 15-Fish/sea food | anchovy, squid, shrimp, mackerel, mussel, octopus, canned sardine, cuttlefish, canned tuna |
| 16-Condiments | garlic, anise, cinnamon, capers, caraway, cumin, turmeric, thyme, fennel seed, harissa**^c^**, |
|  | homemade harissa, ketchup, Knorr® chicken broth, laurel, mint dry, mustard, onion, |
|  | black pepper, added salt (iodised), canned tomato paste, variant, vinegar, yeast cake, baker's yeast |
| 17-Olives/nuts/seeds | almond nuts, peanut, salted peanut, roasted nuts, cashew nut, hazelnut, walnut, olive, green olive, |
|  | pinion, pistachio roasted, sesame seeds, sunflower seeds |
| 18-Olive oil | virgin olive oil |
| 19-Butter/margarine | butter, smen**^d^**, margarin, mayonnaise |
| 20-Processed meat | turkey ham, turkey salami, dried meat |

**^a^** Leben: fermented milk

**^b^** Chamia: typical oriental sweet preparation of sweetened sesame seeds paste often also including dried fruits or nuts.

**^c^** Harissa: typical Tunisian very spicy chili preparation

**^d^**  Smen: salted fermented butter
